# Why do children and adolescents with epilepsy disclose or not disclose their condition to their friends?

**DOI:** 10.1007/s00431-020-03661-0

**Published:** 2020-05-05

**Authors:** Sarah Jeschke, Sarah Woltermann, Martina Patrizia Neininger, Josefine Pauschek, Wieland Kiess, Thilo Bertsche, Astrid Bertsche

**Affiliations:** 1 Neuropaediatrics, University Hospital for Children and Adolescents, Ernst-Heydemann-Str. 8, 18057 Rostock, Germany; 2 Center for Pediatric Research, University Hospital for Children and Adolescents, Liebigstraße 20a, 04103 Leipzig, Germany; 3 Drug Safety Center and Clinical Pharmacy, Institute of Pharmacy, Medical Faculty, Leipzig University, Bruederstraße 32, 04103 Leipzig, Germany

**Keywords:** Epilepsy, Children, Friends, Communication, Stigmatization, Survey

## Abstract

A diagnosis of epilepsy substantially influences the lives of affected children and adolescents, and concealing the diagnosis can generate additional stress. However, little is known about whether children and adolescents communicate their diagnosis to their friends. We performed a survey at two German university hospitals. Epilepsy patients aged 6–18 years were asked why they did or did not disclose their condition to their friends. A total of 101 patients (44 female, 57 male) were interviewed. Twenty-one (21%) informed all their friends about their epilepsy, 63 (62%) informed only certain friends, and 3 (3%) did not specify. Fourteen (14%) did not inform any friends. Their reasons for informing their friends were trust in friends (47/87; 54%); questions from friends, e.g., about missed school days (29/87; 33%); a wish for friends to be informed in case of an emergency (15/87; 17%); and a desire to live openly with the condition (8/87; 9%). The reasons for not informing friends were fear of stigmatization/shame (4/14; 29%), discouragement from parents (3/14; 21%), and a wish for confidentiality (3/14; 21%).

*Conclusion*: Most children and adolescents inform their friends about their epilepsy. Fear of stigmatization is the main reason for not informing friends about this condition.

**Table:** 

**What is Known** *• An epilepsy diagnosis is a life-changing event for affected children and adolescents.* *• Having to conceal the diagnosis can be very stressful.*	
**What is New** *• Most pediatric epilepsy patients informed at least some close friends about their diagnosis motivated by trust in friends; questions from friends,* e.g.*, about missed school days; and a wish for friends to be informed in case of an emergency.* *• Fear of stigmatization/shame, discouragement from parents, and a wish for confidentiality were the motivations for concealing the disease.*

**Electronic supplementary material:**

The online version of this article (10.1007/s00431-020-03661-0) contains supplementary material, which is available to authorized users.

## Introduction

Beginning at school age, children’s relationships with their friends become increasingly important. This is equally true for children with a chronic condition such as epilepsy. Epilepsy has an enormous influence on the daily lives of children and adolescents affected by this condition [10, 21]. In an earlier survey, a quarter of pediatric epilepsy patients reported that they thought they were generally worse off than healthy children [18]. A number of healthy adolescents still have reservations about, for example, being in a romantic relationship with a person with epilepsy [12]. Such reservations could prompt pediatric epilepsy patients to conceal their condition, but having to conceal something as important as a chronic condition can put enormous stress on patients. Data on whether children and adolescents with epilepsy communicate their condition to their friends and what motivates them to disclose or conceal their condition are scarce. A review published in 2015 [5] found only one study from 1968 [13] that examined children’s and adolescents’ disclosure of an epilepsy diagnosis as its main focus. The other studies the authors identified included disclosure as either a subtheme of a larger study or a topic that incidentally emerged in interviews focused on other questions. A study published in 1979 showed that adolescents who had better seizure control communicated their condition less openly and, unexpectedly, had poorer self-image and poorer expectations for the future [11]. In contrast, a recent study showed that adolescents with type 1 diabetes communicated their condition more openly if they had a better health status [22].

A qualitative study published in 2015 in which interviews were conducted with 29 children with epilepsy identified several challenges that might prevent children with epilepsy from disclosing their condition, such as the wish for normalcy [2], which is an important factor in the quality of life of children and adolescents with epilepsy [7]. Interviews with parents indicated that factors such as the wish for normalcy were reasons for not disclosing their child’s condition [3]. In our study, we aimed to explore why children and adolescents did or did not disclose the fact that they had epilepsy to their friends.

## Methods

### Setting and patients

This prospective observational study was performed at the neuropediatric departments of two university hospitals in Germany over 6 months in 2018 and 2019. After approval by the responsible ethics committees, patients aged 6–18 years with a diagnosis of epilepsy were consecutively invited to participate in an interview. The patients and their parents were approached during a hospital stay or appointment at the outpatient clinic. Patients who were accompanied by persons who were not their legal guardians were not included in the study, and children who were unable to understand or answer the questions were excluded. Written informed consent to participate voluntarily in the study was obtained from all participating children and their parents.

### Data assessment

A questionnaire was developed by an expert panel consisting of child neurologists, pharmacists, and a child and adolescent psychotherapist in advanced postgraduate training. The questionnaire was piloted with five children. Based on the pilot survey, the clarity, completeness, comprehensibility, and practicability of the questionnaire were improved. The questionnaire was used for the interview to ensure that all participants received the same questions in the same order. To obtain the best possible insight into the patients’ perceptions, open questions were used. The children and adolescents were interviewed without their parents if the patients and parents agreed. Parents who wanted to stay with their children were instructed not to interfere. There were no time limits for the participants’ answers. The questions were asked and the answers were recorded by a child and adolescent psychotherapist in advanced postgraduate training and a medical student supervised by her.

The answers were clustered by the expert panel for structured data evaluation.

The following questions were asked:

1. Did you tell your friends about your condition? If yes, did you tell all your friends or just special ones?
2. For those patients who had disclosed their condition to their friends:

- Why did you tell your friends about your condition?
- How did your friends react when you told them you have epilepsy?
- What do you believe they think about your condition?

3. For those patients who had not disclosed their condition to their friends:

- Did you have a special reason for not informing your friends about your condition?
- What do you tell your friends if you have a medical appointment?
- How do you think your friends would react if you told them you have epilepsy?
- What do you believe they would think of you if they knew you had epilepsy?
- Would you like to confide in one of them about your condition someday?

Furthermore, data on epilepsy diagnosis and sociodemographic data were retrieved from the patients’ medical records.

### Statistics

Calculations were performed using SPSS (Statistical Package for the Social Sciences, Version 20, IBM, USA). Frequencies are reported as the absolute and relative numbers. Continuous data are presented as the median and quartiles. For further statistical analysis, we applied chi-square tests, Fisher’s exact tests, or Mann–Whitney *U* tests, as appropriate. A *p* value ≤ 0.05 was considered to indicate significance.

## Results

A total of 101 children and adolescents participated in the survey. The patients’ characteristics are displayed in Table 1. Of the 101 children and adolescents, 87 (86%) reported that they had informed their friends about their condition and 14 (14%) said they had not. Regarding the characteristics of patients who had disclosed their condition and those who had not, no significant differences were found. Twenty-one (21%) patients indicated that they had informed all of their friends about their condition, whereas 63 (62%) participants reported that they had told only certain friends, such as best friends, and 3 (3%) did not specify.

**Table 1:** Patients’ characteristics. Frequencies are reported as the absolute and relative numbers. Continuous data are given as the medians and quartiles. For statistical analysis, we applied chi-square tests, Fisher’s exact tests, or Mann–Whitney *U* tests, as appropriate. A *p* value ≤ 0.05 was considered to indicate significance

Characteristic	Total	Participants who disclosed their epilepsy to their friends	Participants who did not disclose their epilepsy to their friends	*p* value
Number of participants, *N*	101	87	14	
Male, *N* (%)	57 (56%)	48 (55%)	9 (50%)	n.s.
Female, *N* (%)	44 (44%)	39 (45%)	5 (50%)	
Age median [Q25/Q75]	11 [8/14]	11 [9/14]	9 [8/12]	n.s.
Diagnosis, *N* (%)				
Rolandic epilepsy	20 (20%)	18 (21%)	2 (14%)	n.s.
Other epilepsy with focal seizures	40 (40%)	36 (41%)	4 (29%)	n.s.
Absence epilepsy	20 (20%)	18 (21%)	2 (14%)	n.s.
Other epilepsy with generalized seizures	14 (14%)	10 (11%)	4 (29%)	n.s.
Unclassified epilepsy	7 (7%)	5 (6%)	2 (14%)	n.s.
Seizure occurrence, *N* (%)				
Seizures within the last 12 months	55 (54%)	49 (56%)	6 (42%)	n.s.
Education, *N* (%)				
Primary school	39 (39%)	31 (36%)	8 (57%)	n.s.
Middle school	23 (23%)	22 (25%)	1 (7%)	n.s.
Grammar school	17 (17%)	16 (18%)	1 (7%)	n.s.
Vocational school	3 (3%)	3 (3%)	0 (0%)	n.s.
Special needs school	19 (19%)	15 (17%)	4 (29%)	n.s.

### Participants who disclosed their epilepsy to their friends

#### “Why did you tell your friends about your condition?”

The reasons mentioned by the 87 participants for disclosing their condition (open question with multiple possible answers) were trust in their friends (47; 54%); questions from their friends, e.g., about missed school days (29; 33%); a wish for their friends to know about the condition in case of an emergency (15; 17%); and a desire to live openly with the condition (8; 9%). Eight (9%) participants did not give a reason. Details can be found in Table S1 in the supplementary material.

#### “How did your friends react when you told them you have epilepsy?”

According to the 87 participants who had disclosed their condition, the dominant reactions friends showed when they learned about the epilepsy were empathy (29; 33%), e.g., “They said they would care for me if I needed them”; a request for information (21; 24%), e.g., “They asked if it can be fatal”; astonishment (6; 7%), e.g., “They were very startled”; and no reaction at all (2; 2%). Twenty-nine (33%) participants did not specify.

#### “What do you believe they think about your condition?”

When they were asked what they assumed their friends thought about their condition, the 87 participants who had informed their friends about their epilepsy most commonly assumed that their friends worried about them (12; 14%). More information about answers to this question is displayed in Table S2 of the supplementary material.

### Participants who did not disclose their epilepsy to their friends

#### “Did you have a special reason for not informing your friends about your condition?”

Of the 14 participants who said that their friends did not know about their epilepsy, 4 (29%) participants said they had no special reason for not informing their friends, and 10 (71%) answered they did have reasons. The reported reasons are displayed in Table 2.

**Table 2:** Reasons children and adolescents reported for not informing their friends about their condition (*n* = 14 participants)

Category	Examples
Fear of stigmatization/shame (4; 29%)	“I do not dare. I am afraid they will laugh at me.”
Discouragement from parents (3; 21%)	“Mom and Dad do not allow me.”
Wish for confidentiality (3; 21%)	“I do not want to tell anyone.”
	“I do not want to.”
Reason not given (4; 29%)	

#### “What do you tell your friends if you have a medical appointment?”

Of the participants who had not informed their friends about their condition, 7 (50%) stated that they told their friends when they had a (medical) appointment but remained unspecific about the reason. Three (21%) said that they did not tell them about appointments and/or that nobody asked them about it. Four (29%) answered that they did not know what they disclosed to their friends.

#### “How do you think your friends would react if you told them you have epilepsy?”

When asked how they believed their friends would react upon learning about their condition, 5 (36%) anticipated an uncomfortable reaction, e.g., “They could laugh at me” or “They would keep asking questions”; 4 (29%) assumed that their friends would show empathy and offer support, e.g., “They would worry about me” or “They would accept it”; and 5 (36%) had no idea.

#### “What do you believe they would think of you if they knew you had epilepsy?”

Of the 14 participants, 4 (29%) assumed that their friends would have neutral thoughts, 3 (21%) assumed that their friends worry about them, and 3 (21%) assumed that their friends would have negative thoughts if they knew the participant had epilepsy. The assumed thoughts are displayed in Table 3.

**Table 3:** Responses of the participants who concealed their epilepsy to the question, “What do you believe they would think of you if they knew you had epilepsy?” (*n* = 14 participants)

Category	Reported assumed thoughts
Neutral thoughts (4; 29%)	“They would not care.”
	“He has laughing fits, that is it.”
	“Nothing bad.”
	“That is o.k.”
Worries about the patient (3; 21%)	“They would not be happy; they might worry.”
	“That it is a bad condition because they do not like me being away.”
	“They would fear for me.”
Negative thoughts (3; 21%)	“They might think that I am infecting them if I touched them.”
	“They might be angry.”
	“They would make fun of me.”
Do not know (4; 29%)	

#### “Would you like to confide in one of them someday about your condition?”

Of the 14 participants who had not informed their friends about their condition, 2 (14%) said they would like to confide in a friend someday, 9 (64%) said they would not, and 3 (21%) did not know.

## Discussion

We conducted a survey of children and adolescents with epilepsy to ask whether and why they had disclosed their epilepsy to their friends. It is encouraging that such a high percentage, i.e., 86%, of the children and adolescents had told their friends they had epilepsy. In contrast, in a study of children with asthma, only 26% had disclosed their condition [19]. It is even more encouraging that most children who had disclosed their epilepsy reported positive experiences. Most young people with epilepsy in this study chose to tell close friends about their diagnosis. This is quite understandable, since having a chronic condition can be experienced as very sensitive information by the affected person. Because a seizure can occur at any time, however, the wish to conceal the information that they have epilepsy, from classmates, for example, can negatively influence the quality of life of the children and adolescents with epilepsy [14].

In the present study, the most commonly reported reason for disclosing the condition was trust in friends. Epilepsy is such an important part of the daily life of most affected patients that it can be very difficult if they feel they cannot speak to anyone about it. It is encouraging that the participants in our study reported that their friends showed interest and empathy.

A number of children said they informed their friends about the epilepsy so that their friends would know about it in case of an emergency. This approach reflects thoughtful management of the condition. Patient safety can be enhanced if children inform their teacher, for example. However, as the emotional aspect of a conversation is also important, children and adolescents should be encouraged to communicate not only the technical aspects of their condition, such as the management of an acute seizure or medication. To develop appropriate strategies for the disclosure of their epilepsy, young people could be supported by the How2tell multimedia self-management resource for people with epilepsy [8].

In accordance with an earlier study [2], approximately one-third of our patients who concealed their condition anticipated a negative reaction. Interestingly, the other two-thirds, however, anticipated a positive reaction or had no idea how their friends would react but nevertheless did not disclose that they had epilepsy for various reasons. Another challenge identified in the study reported by Benson et al. [2] was that pediatric epilepsy patients perceived their condition to be very private. Participants in our study also reported a wish for confidentiality. In addition, some children even said their parents forbade them to communicate about the condition. This desire of parents to keep epilepsy a secret has also been described in earlier studies [16]. As this parental wish leaves children and adolescents alone with their condition, parents should be encouraged not to make such a demand. Open communication about epilepsy within the family improves quality of life for children with epilepsy [17]. Because having friends outside the family becomes increasingly important as a child grows older and gains more autonomy, communication outside the family should also be encouraged.

Participants in the study by Benson et al. [2] complained about the lack of public knowledge about epilepsy and the public perception of epilepsy as something “weird.” Such perceptions and lack of knowledge lead to a fear of stigmatization, which was one of the factors identified in our study that prevented children and adolescents from disclosing their epilepsy diagnosis. A higher perception of stigma correlates with noncommunication about epilepsy outside the family [4]. In an earlier study, 10% of the participants thought that epilepsy was contagious [18]. Participants in the present study described that their friends thought they had a transmittable condition (e.g., “First, they thought I had rabies…”). Such perceptions can lead to an even greater fear of stigmatization. In another study, adults who did not talk about their condition perceived significantly higher stigma levels [15]. Greater need for information and support was associated with a greater perception of stigmatization of pediatric patients [1]. Stigmatization is not only feared by patients with epilepsy but is present in the population. Between 2005 and 2013, the percentage of people who said they would be nervous around people with epilepsy increased [6]. In a German survey of more than 1000 students aged approximately 14 years, one-fifth of the participants reported that they would not want to go on a romantic date with a person with epilepsy [12]. Parents of children with epilepsy and developmental disorders reported discrimination against their children [21]. As peer support is associated with a better quality of life for children with epilepsy [9], it seems necessary to reduce stigmatization and improve support by educating the general public, including children and adolescents. Campaigns such as the Epilepsy Foundation public awareness campaigns can help to reduce stigmatization [20].

### Limitations

The study was performed at the neuropediatric departments of two German university hospitals. The results might be different for other regions of Europe or the world.

## Conclusion

It is very encouraging that most children and adolescents with epilepsy disclosed the condition to their friends and reported positive experiences. The main motivation reported for not informing friends was a fear of stigmatization. To improve the emotional well-being, participation, and safety of pediatric epilepsy patients, they should be encouraged to communicate openly about their condition. Health care and education professionals could address the positive experiences of patients who have disclosed their condition to their friends in their communication with children and adolescents suffering from epilepsy and offer their support.

## Electronic supplementary material

ESM 1 (DOCX 21 kb)

ESM 2 (DOCX 23 kb)
