# Supplementary material for: Why do children and adolescents with epilepsy disclose or not disclose their condition to their friends?
Source: Eur J Pediatr. 2020 May 5;179(10):1627–33. doi: 10.1007/s00431-020-03661-0 (PMC7479002; doi:10.1007/s00431-020-03661-0)
Supplement: Supplementary file 1 — (DOCX 21 kb) [file 431_2020_3661_MOESM1_ESM.docx]

Table S1: Children’s and adolescents’ reasons for disclosing their condition to their friends (open question with multiple possible answers possible; n=87 participants)

| Category | Examples |
| --- | --- |
| Trust in friends (47; 54%) | “Because they say, ‘Ok, you are still normal’; they always listen and care for me.” |
| Questions from friends  (29; 33%) | “They asked me why I missed school.” |
|  | “They asked me what was wrong with  me.” |
|  | “The seizure was at a children's birthday party, I then told all the guests about it, because they were wondering what was going on.” |
|  | “Because I was asked about a doctor's appointment.” |
| Wish for friends to be informed in case of an emergency  (15; 17%) | “So that they are informed, can help in an emergency and know triggers, can put themselves in my situation.” |
| Wish to live openly with the condition  (8; 9%) | “I wanted to be open about it.” |
|  | “I did not want to have secrets.” |
| Reason not given  (8; 9%) | “No, there is no such thing.” |
