# Supplementary material for: Why do children and adolescents with epilepsy disclose or not disclose their condition to their friends?
Source: Eur J Pediatr. 2020 May 5;179(10):1627–33. doi: 10.1007/s00431-020-03661-0 (PMC7479002; doi:10.1007/s00431-020-03661-0)
Supplement: Supplementary file 2 — (DOCX 23 kb) [file 431_2020_3661_MOESM2_ESM.docx]

Table S2 Thoughts that participants who informed their friends about their epilepsy assumed their friends had about their condition (n=87 participants)

| Category | Examples of reported assumed thoughts |
| --- | --- |
| Worries about the patient or compassion (12; 14%) | “They hope nothing happens to me.” |
|  | “They worry that I might keel over and go into convulsions.” |
|  | “One of them said, ‘Oh, poor you’, so they think of me, but I do not know what they think.” |
|  | “What? That cannot be.” |
|  | “They are afraid for me. They are afraid of the condition. They wonder what to do.” |
| Questions about the condition/lack of understanding (10; 11%) | “What happens with this condition? Can that happen to me?” |
|  | “Is that bad? Can you still play with him?” |
|  | “What is he talking about, and what is wrong with him?” |
|  | “First, they thought I had rabies. Then I told them about the epilepsy, and now they know all about it.” |
|  | “They think that I get electric shocks because it sounded that way once and they overheard it.” |
| Acceptance of patient in spite of the condition (9; 10%) | “That is the way things are. We take care of each other.” |
|  | “He is now impaired and on medication, but we accept that.” |
|  | “They want to stand by me and help me.  It is just there; that is okay. |
|  | “I assume they think that I am still a normal girl and that they should continue to treat me as a normal girl.” |
| No worries about the condition (4; 5%) | “They do not think about it, because it is not even noticeable.” |
|  | “I do not think they think anything bad about it.” |
| Questions about how to deal with the patient (4; 5%) | “Uh-oh, is there anything in particular I need to pay attention to now?” |
|  | “That they need to be careful now.” |
|  | “How can I help?” |
| Thoughts about recovery (3; 3%) | “Hopefully it will get better soon.” |
|  | “They think that the condition could go away again.” |
| Negative thoughts (3; 3%) | “They think that the condition is quite annoying.” |
|  | “If there are days when I have to be picked up, they say, ‘She's playing hooky’, so actually they are negative about it.” |
| Worries that the patient might feel stigmatized (3; 3%) | “I would like to ask you so many questions, but I did not dare.” |
|  | “They think that no jokes are to be made about the condition and that it should not be a silly topic of conversation, that seriousness in dealing with it is necessary.” |
| Other thoughts (4; 5%) | “That sometimes I am not truly there; I am gone for a little while.” |
|  | “That the hospital stays and doctor's appointments are related to it.” |
|  | “I told him a lot about it, including that sometimes I have to go to university hospital. In addition, he already knew that.” |
|  | “They think it is something bad.” |
| Do not know (35; 40%) |  |
